# Supplementary material for: Revisiting species and areas of interest for conserving global mammalian phylogenetic diversity
Source: Nat Commun. 2021 Jun 17;12:3694. doi: 10.1038/s41467-021-23861-y (PMC8211746; doi:10.1038/s41467-021-23861-y)
Supplement: Supplementary file 1 — Supplementary Information [file 41467_2021_23861_MOESM1_ESM.pdf]

Nature Communications

SUPPLEMENTARY INFORMATION

Revisiting species and areas of interest for conserving global mammalian phylogenetic diversity

Marine Robuchon, Sandrine Pavoine, Simon Véron, Giacomo Delli, Daniel P. Faith, Andrea Mandrici, Roseli Pellens, Grégoire Dubois, Boris Leroy

Supplementary Table 1 Number of grid cells in common (%) between hotspots for the 12 scores calculated by grid cell. SR = species richness, TSR = threatened species richness, ESR = endemic species richness, SWE = species-weighted endemism, PD = phylogenetic diversity, TPD = threatened phylogenetic diversity, EPD = endemic phylogenetic diversity, PWE = phylogenetic-weighted species endemism, HEDGE = species richness of TOP HEDGE species, LEDGE = species richness of TOP LEDGE species, GexpPD = expected gain in phylogenetic diversity if all species present in the cell are saved from extinction, and LexpPD = expected loss in phylogenetic diversity if all species present in the cell become extinct.

	SR	TSR	ESR	SWE	PD	TPD	EPD	PWE	HEDGE	LEDGE	GexpPD	LexpPD
**SR**	100											
**TSR**	41	100										
**ESR**	29	42	100									
**SWE**	35	45	67	100								
**PD**	87	37	26	31	100							
**TPD**	47	74	33	40	45	100						
**EPD**	32	42	76	68	29	38	100					
**PWE**	42	51	64	80	36	46	67	100				
**HEDGE**	39	83	43	44	35	65	41	50	100			
**LEDGE**	79	33	24	27	78	43	26	32	30	100		
**GexpPD**	22	52	28	32	25	45	31	38	52	13	100	
**LexpPD**	85	34	24	29	80	40	27	35	33	84	12	100

Supplementary Table 2 Number of species in common (%) between the 1369 TOP 25% EDGE species, the 1369 TOP 25% HEDGE species and the 1369 TOP 25% LEDGE species.

	TOP 25 % EDGE	TOP 25 % HEDGE	TOP 25 % LEDGE
**TOP 25 % EDGE**	100		
**TOP 25 % HEDGE**	87	100	
**TOP 25 % LEDGE**	31	20	100

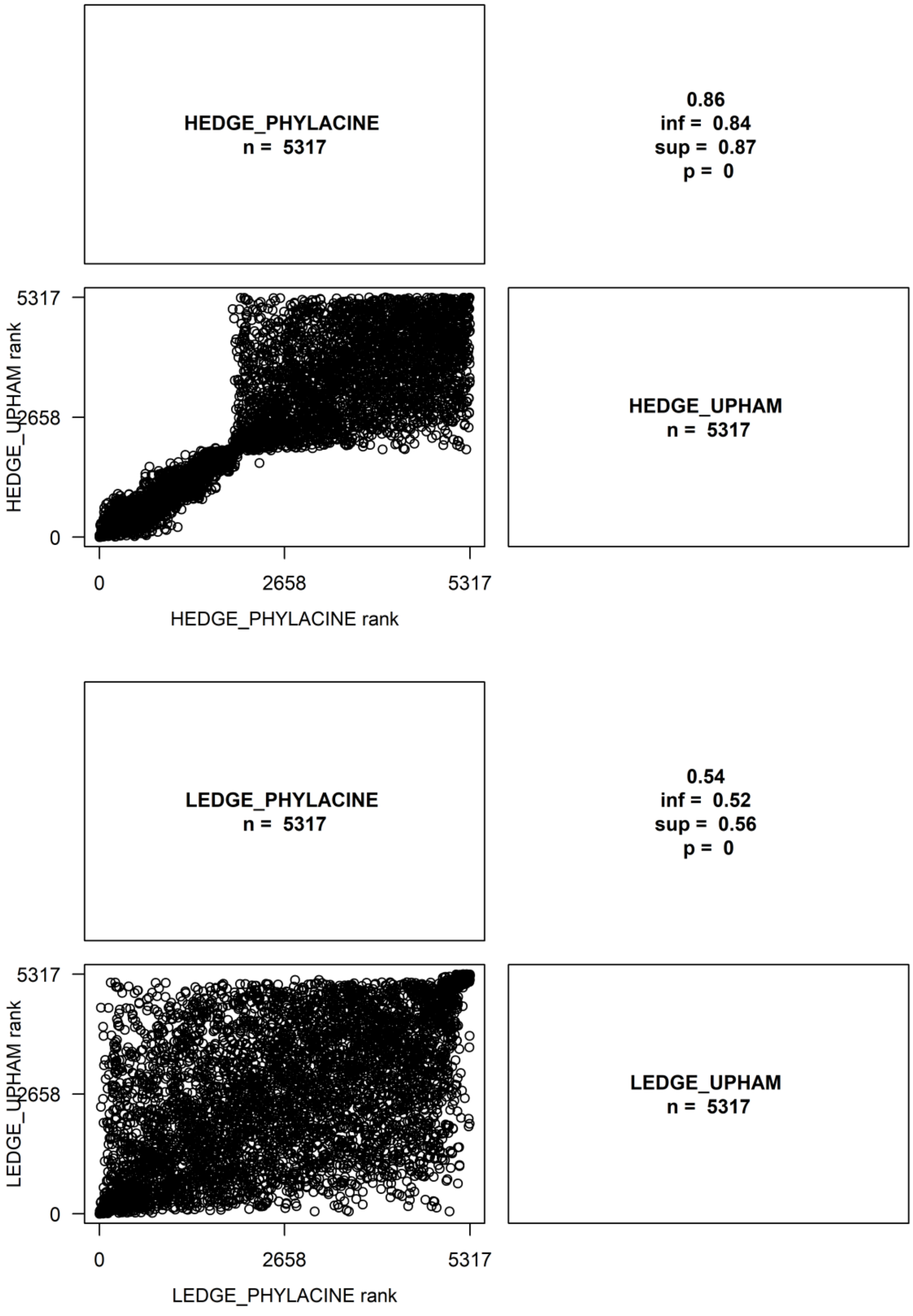

Supplementary Figure 1 Two-sided Spearman correlations tests between scores based on the phylogeny from PHYLACINE and those based on the phylogeny from Upham et al. (2019) for HEDGE scores (top graphs) and LEDGE scores (bottom graphs) over the distinct species. Numbers above the diagonal correspond to, from top to bottom, Spearman correlation coefficients, inferior and superior 95% confidence intervals for the correlation coefficient, and p-value. n correspond to the number of mammal species common to the two phylogenies.

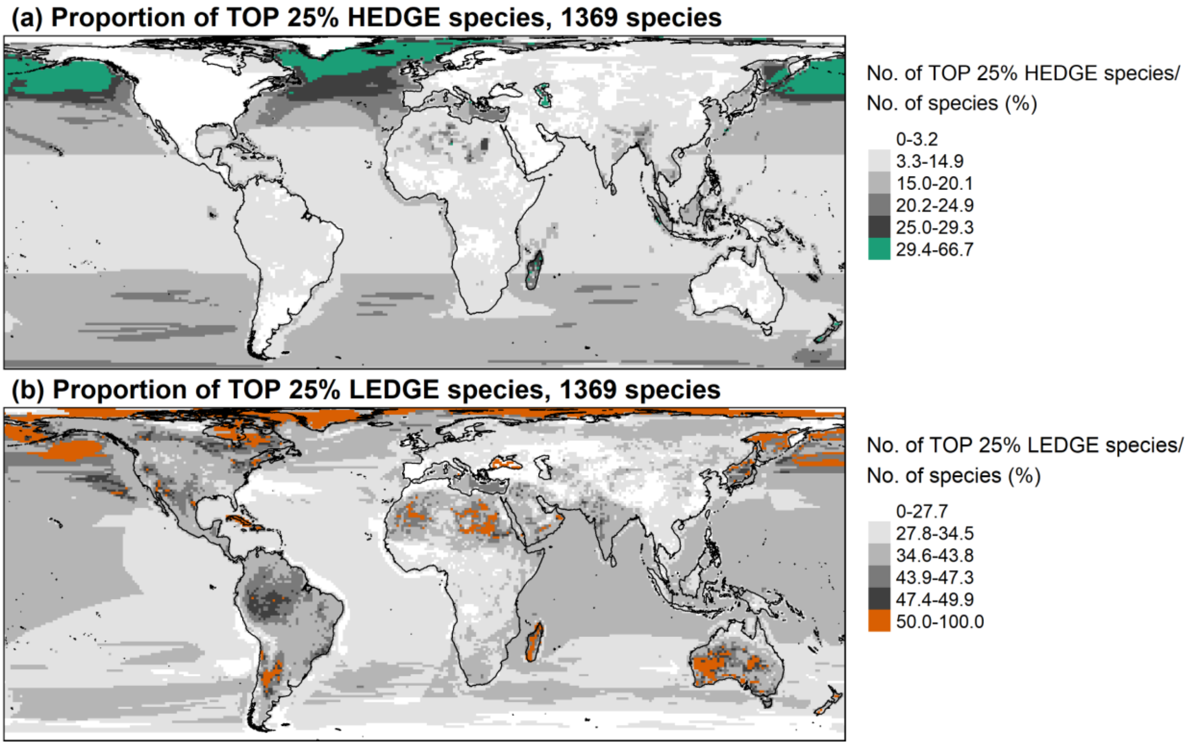

Supplementary Figure 2 Spatial patterns and hotspots of proportion of species richness for the TOP 25% HEDGE species (a) and the TOP 25% LEDGE species (b). Hotspots, in colour, represent the 2.5% richest cells.

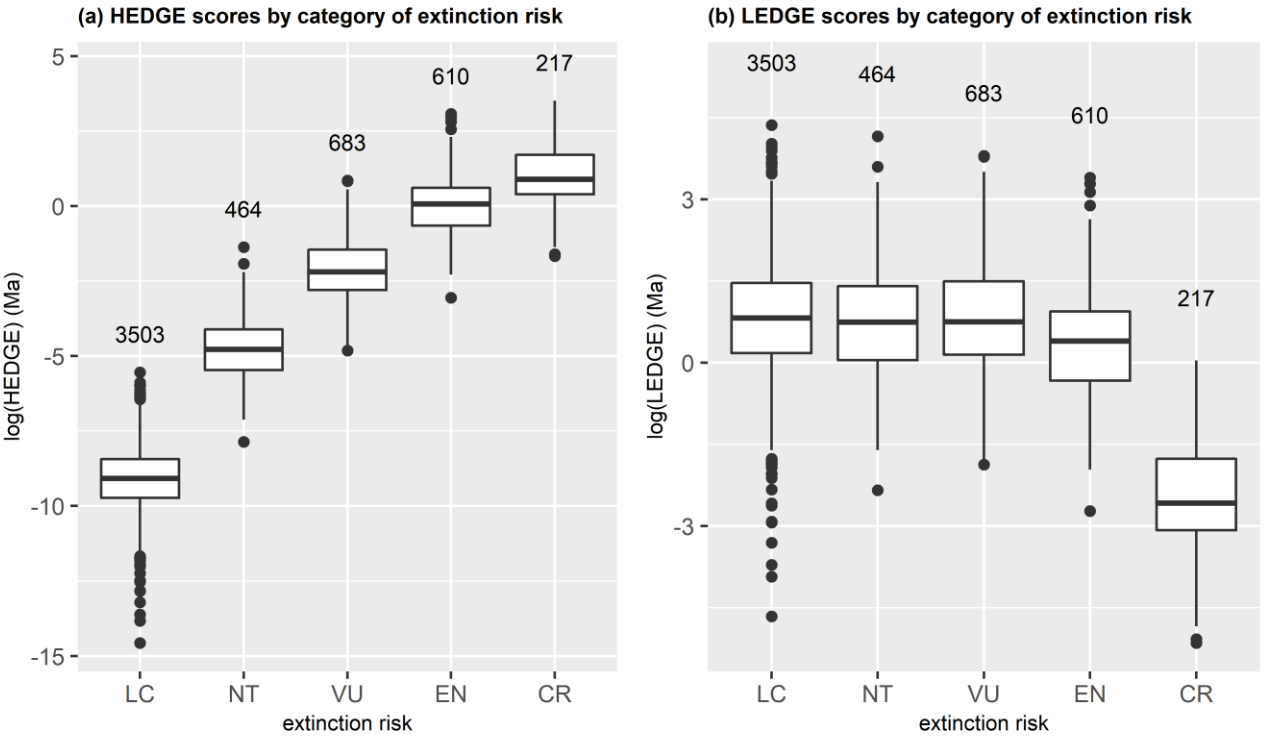

Supplementary Figure 3 Distribution of HEDGE scores (a) and LEDGE scores (b) by category of extinction risk (LC = Least Concern, NT = Near Threatened, VU = Vulnerable, EN = Endangered, CR = Critically Endangered) for the 5477 extant mammal species. The boxes represent the interquartile range (minima = 25th percentile, maxima = 75th percentile). The whiskers extend from the smallest value within 1.5 times the interquartile range below the 25th percentile to the largest value within 1.5 times the interquartile range above the 75th percentile. The points represent ouliers, i.e. values between 1.5 and 3 times the interquartile range. The horizontal bold black lines represent the median values. The numbers above the boxplots represent the number of species by category of extinction risk. Source data are provided as a Source Data file

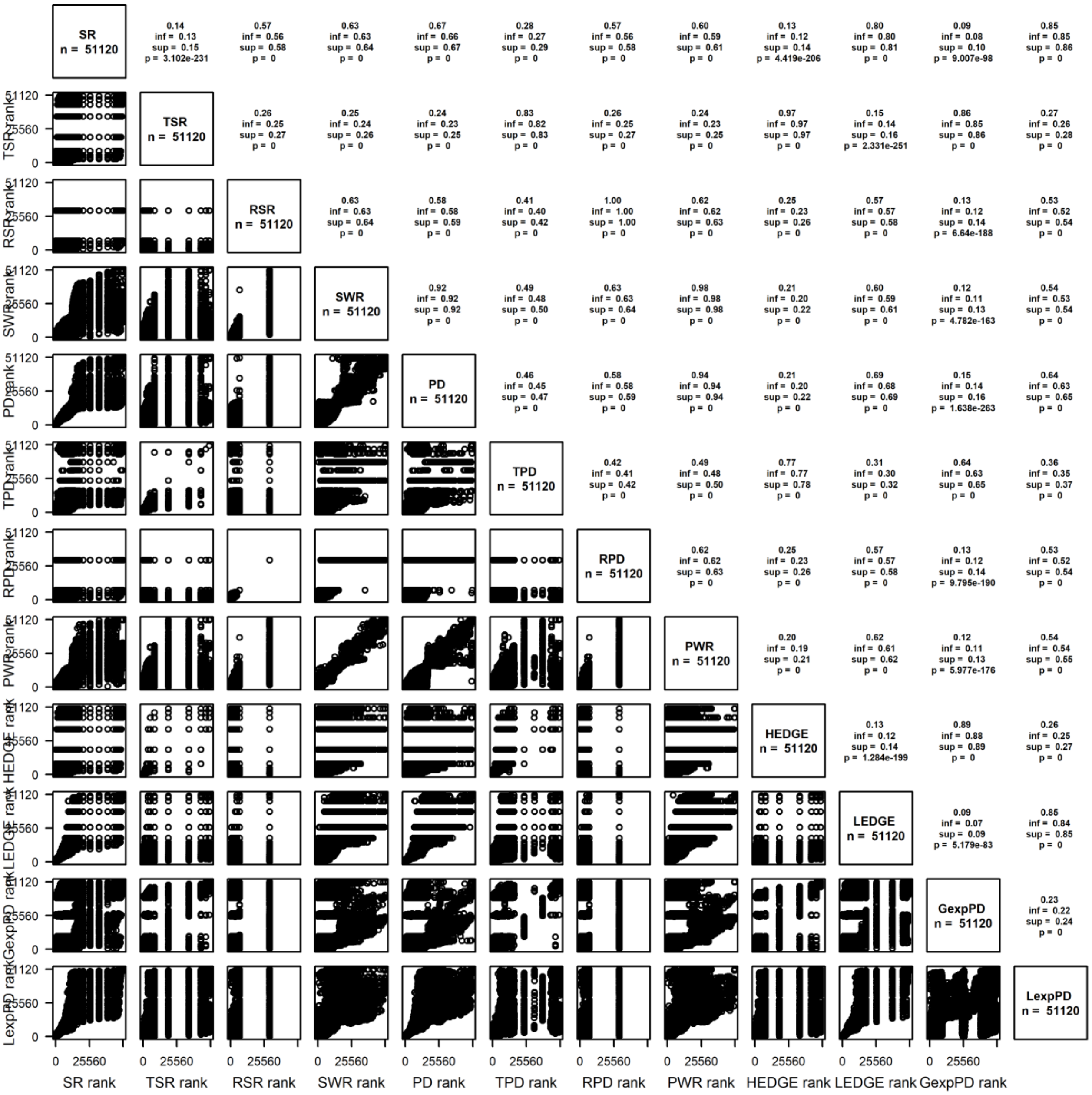

Supplementary Figure 4 Two-sided Spearman correlations tests between the 12 spatial scores over the distinct grid cells. SR = species richness, TSR = threatened species richness, ESR = endemic species richness, SWE = species-weighted endemism, PD = phylogenetic diversity, TPD = threatened phylogenetic diversity, EPD = endemic phylogenetic diversity, PWE = phylogenetic-weighted species endemism, HEDGE = species richness of TOP HEDGE species, LEDGE = species richness of TOP LEDGE species, GexpPD = expected gain in phylogenetic diversity if all species present in the cell are saved from extinction, and LexpPD = expected loss in phylogenetic diversity if all species present in the cell become extinct. Numbers above the diagonal correspond to, from top to bottom, Spearman correlation coefficients, inferior and superior 95% confidence intervals for the correlation coefficient, and p-value. n correspond to the number of grid cells.

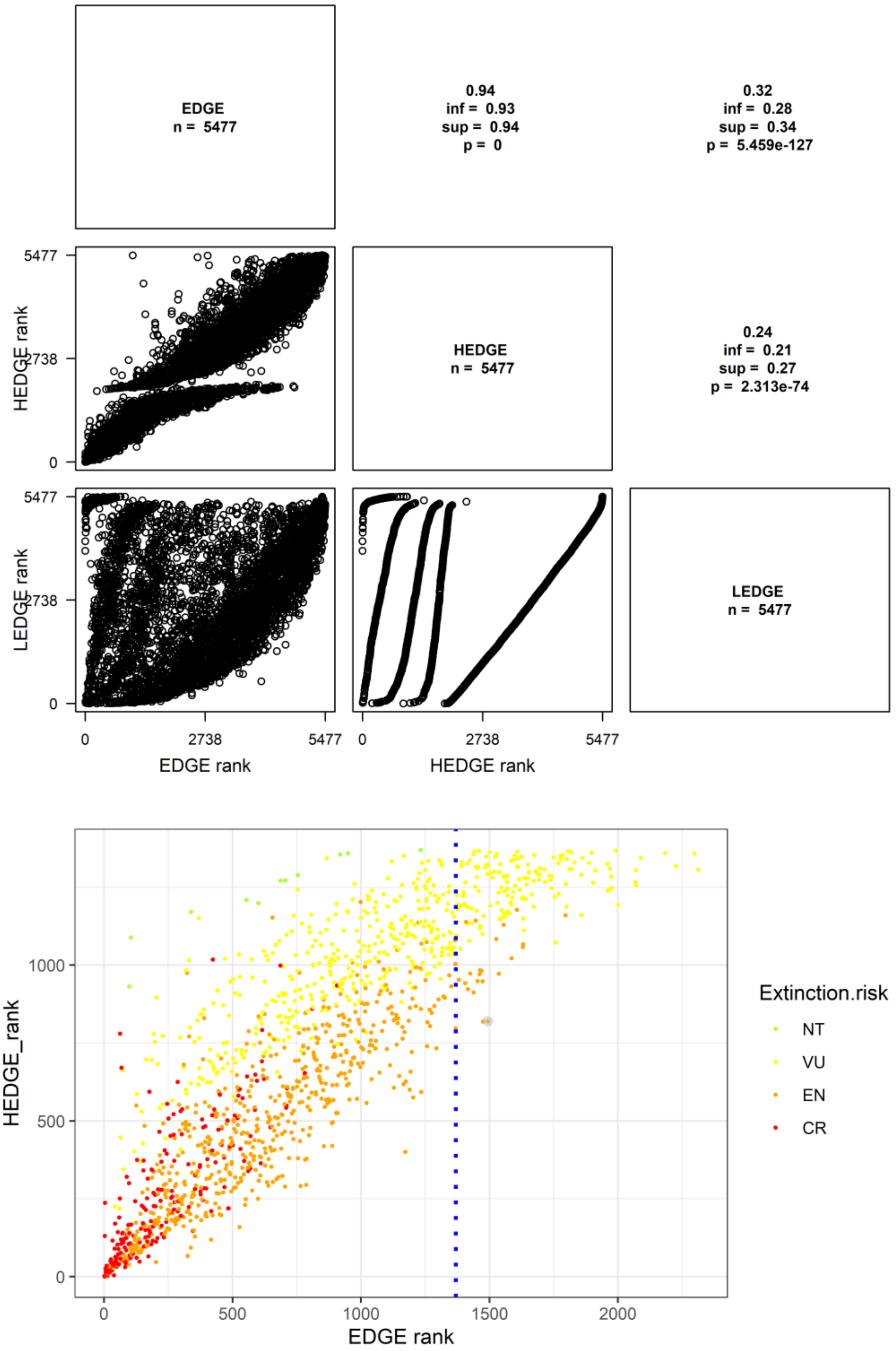

Supplementary Figure 5 At the top: two-sided Spearman correlations tests between EDGE, HEDGE and LEDGE scores over the distinct species for all mammal species. Numbers above the diagonal correspond to, from top to bottom, Spearman correlation coefficients, inferior and superior 95% confidence intervals for the correlation coefficient, and p-value. n correspond to the number of mammal species. **At the bottom: zoom showing the relation between EDGE and HEDGE ranks for the TOP 25% HEDGE species**. Colours represent the extinction risk of the species (LC = Least Concern, NT = Near Threatened, VU = Vulnerable, EN = Endangered, CR = Critically Endangered). The grey-shaded surrounded point represents the endangered species *Pteropus melanopogon* (HEDGE rank = 820, EDGE rank = 1494).

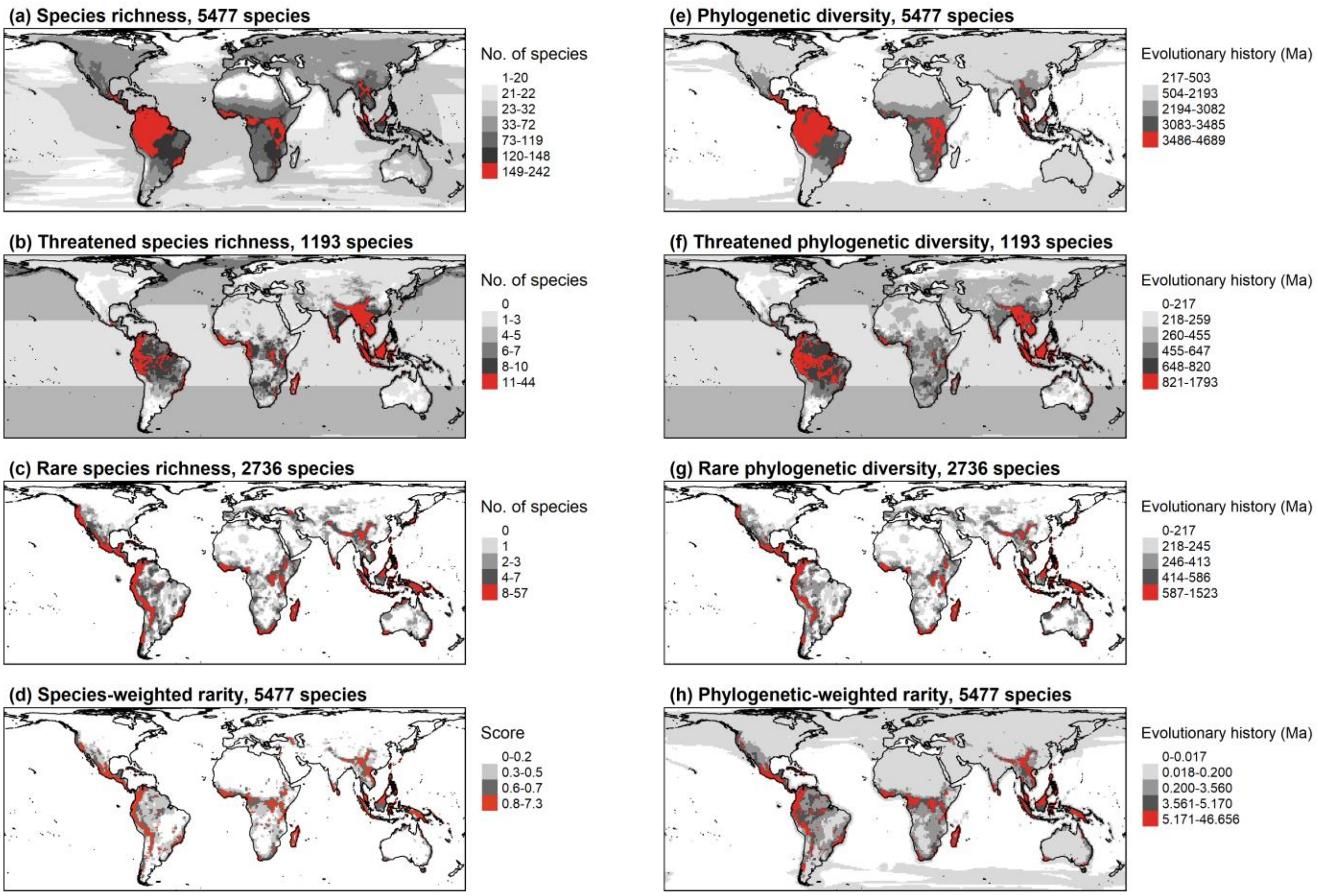

Supplementary Figure 6 Spatial patterns and hotspots of species richness (a), threatened species richness (b), rare species richness (c), species-weighted rarity (d), phylogenetic diversity (e), threatened phylogenetic diversity (f), rare phylogenetic diversity (g) and phylogenetic-weighted rarity (h). Hotspots, in red, represent the 2.5% cells with the highest values.

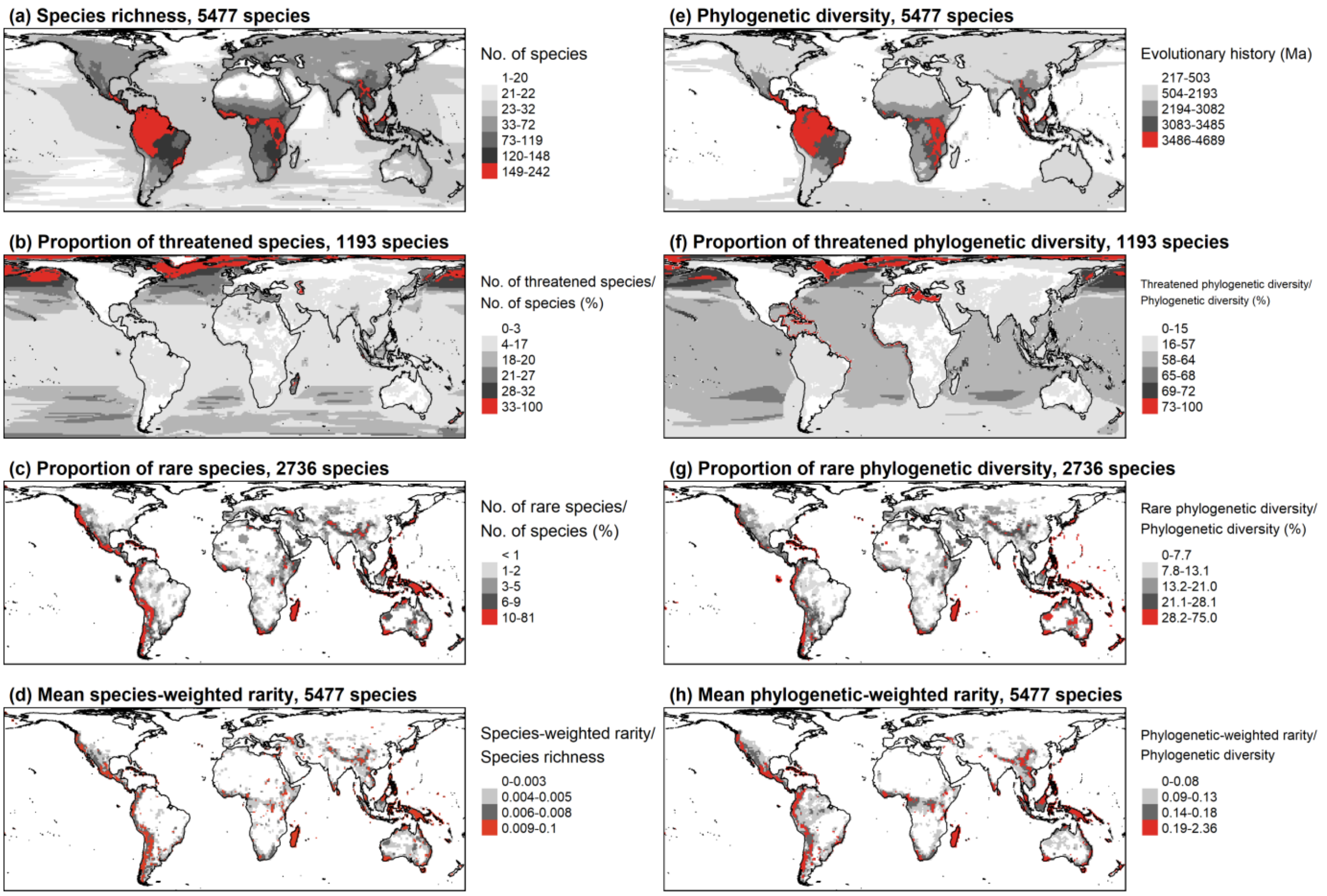

Supplementary Figure 7 Spatial patterns and hotspots of species richness (a), proportion of threatened species (b), proportion of rare species (c), mean species-weighted rarity (d), phylogenetic diversity (e), proportion of threatened phylogenetic diversity (f), proportion of rare phylogenetic diversity (g) and mean phylogenetic-weighted rarity (h). Hotspots, in red, represent the 2.5% cells with the highest values.
