# Supplementary material for: Revisiting species and areas of interest for conserving global mammalian phylogenetic diversity
Source: Nat Commun. 2021 Jun 17;12:3694. doi: 10.1038/s41467-021-23861-y (PMC8211746; doi:10.1038/s41467-021-23861-y)
Supplement: Supplementary file 3 — Reporting Summary [file 41467_2021_23861_MOESM3_ESM.pdf]

nature research

Corresponding author(s): Marine Robuchon

Last updated by author(s): May 5, 2021

Reporting Summary

Nature Research wishes to improve the reproducibility of the work that we publish. This form provides structure for consistency and transparency in reporting. For further information on Nature Research policies, see our Editorial Policies and the Editorial Policy Checklist.

Statistics

For all statistical analyses, confirm that the following items are present in the figure legend, table legend, main text, or Methods section.

n/a	Confirmed	
☐	☒	The exact sample size (*n*) for each experimental group/condition, given as a discrete number and unit of measurement
☐	☒	A statement on whether measurements were taken from distinct samples or whether the same sample was measured repeatedly
☐	☒	The statistical test(s) used AND whether they are one- or two-sided<br>*Only common tests should be described solely by name; describe more complex techniques in the Methods section.*
☐	☒	A description of all covariates tested
☐	☒	A description of any assumptions or corrections, such as tests of normality and adjustment for multiple comparisons
☐	☒	A full description of the statistical parameters including central tendency (e.g. means) or other basic estimates (e.g. regression coefficient) AND variation (e.g. standard deviation) or associated estimates of uncertainty (e.g. confidence intervals)
☐	☒	For null hypothesis testing, the test statistic (e.g. F, t, r) with confidence intervals, effect sizes, degrees of freedom and P value noted<br>*Give P values as exact values whenever suitable.*
☒	☐	For Bayesian analysis, information on the choice of priors and Markov chain Monte Carlo settings
☐	☒	For hierarchical and complex designs, identification of the appropriate level for tests and full reporting of outcomes
☐	☒	Estimates of effect sizes (e.g. Cohen's *d*, Pearson's *r*), indicating how they were calculated

Our web collection on statistics for biologists contains articles on many of the points above.

Software and code

Policy information about availability of computer code

Data collection	R version 3.6.3 was used with the package ‘rredlist’ version 0.6.0 to collect data on conservation measures from the IUCN Red List version 2019-2. All other data were manually downloaded.
Data analysis	The processing of the original WPDA dataset was done using GDAL 3.0, GRASS GIS 7.8, PostgreSQL 12 / Postgis 3.0. All other analyses were done using R. The R code to reproduce the analyses is publicly available on the following GitHub repository: https://github.com/MarineRobuchon/consmampd.

For manuscripts utilizing custom algorithms or software that are central to the research but not yet described in published literature, software must be made available to editors and reviewers. We strongly encourage code deposition in a community repository (e.g. GitHub). See the Nature Research guidelines for submitting code & software for further information.

Data

Policy information about availability of data

All manuscripts must include a data availability statement. This statement should provide the following information, where applicable:

- Accession codes, unique identifiers, or web links for publicly available datasets
- A list of figures that have associated raw data
- A description of any restrictions on data availability

This study used datasets that are publicly available from PHYLACINE (https://doi.org/10.5061/dryad.bp26v20) and VertLife (https://data.vertlife.org/). It also uses data from IUCN version 2019-2 that are available from the corresponding author upon request and with permission of IUCN. The coastline dataset used to make Figures. 3, 4, 6, and Supplementary Figures 2, 6 and 7 is available from Natural Earth (https://www.naturalearthdata.com/downloads/50m-physical-vectors/). All datasets generated during this study are publicly available on the following GitHub repository: https://github.com/MarineRobuchon/consmampd. Source data are provided with this paper.

Field-specific reporting

Please select the one below that is the best fit for your research. If you are not sure, read the appropriate sections before making your selection.

☐ Life sciences ☐ Behavioural & social sciences ☒ Ecological, evolutionary & environmental sciences

For a reference copy of the document with all sections, see nature.com/documents/nr-reporting-summary-flat.pdf

Ecological, evolutionary & environmental sciences study design

All studies must disclose on these points even when the disclosure is negative.

Study description	We used available phylogenetic, distribution and extinction risk data for the world's mammals to re-identify species and areas of interest to preserve global mammalian phylogenetic diversity. We then analysed how these species and areas of interest are currently protected.
Research sample	The research sample comprises the world's mammals. Reference phylogenetic, distribution and extinction risk data come from the PHYLACINE database (version 1.2.0).
Sampling strategy	Sample sizes (5,477 mammal species and 51,120 grid cells) were determined by the full taxonomy and the extent & resolution of species range maps available in the original PHYLACINE database (version 1.2.0) used for the analyses
Data collection	Data were collected from publicly available sources
Timing and spatial scale	Data are global and were collected in 2019
Data exclusions	No data were excluded from the analyses
Reproducibility	All the calculations of species and spatial scores can be replicated using the R code available on the following GitHub repository: https://github.com/MarineRobuchon/consmampd
Randomization	We allocated the 5477 mammal species into groups corresponding to their level of extinction risk to analyse how HEDGE and LEDGE scores calculated here vary with extinction risk
Blinding	Blinding is not relevant for this study which focuses on re-identifying species and areas of interest for the conservation of global mammalian phylogenetic diversity

Did the study involve field work? ☐ Yes ☒ No

Reporting for specific materials, systems and methods

We require information from authors about some types of materials, experimental systems and methods used in many studies. Here, indicate whether each material, system or method listed is relevant to your study. If you are not sure if a list item applies to your research, read the appropriate section before selecting a response.

Materials & experimental systems

n/a	Involved in the study	
☒	☐	Antibodies
☒	☐	Eukaryotic cell lines
☒	☐	Palaeontology and archaeology
☒	☐	Animals and other organisms
☒	☐	Human research participants
☒	☐	Clinical data
☒	☐	Dual use research of concern

Methods

n/a	Involved in the study	
☒	☐	ChIP-seq
☒	☐	Flow cytometry
☒	☐	MRI-based neuroimaging
