# Supplementary material for: Revisiting species and areas of interest for conserving global mammalian phylogenetic diversity
Source: Nat Commun. 2021 Jun 17;12:3694. doi: 10.1038/s41467-021-23861-y (PMC8211746; doi:10.1038/s41467-021-23861-y)
Supplement: Supplementary file 4 — Description of Additional Supplementary Files [file 41467_2021_23861_MOESM4_ESM.pdf]

Description of Additional Supplementary Files

File Name: Supplementary Data 1
Description: List of the 5477 extant mammal species (species), their classification (order, family), their real IUCN status (IUCN_status_real), their imputed IUCN status (IUCN_status_imputed), their global endangerment category (GE), their ED scores (ED) and ranks (ED_rank), their EDGE scores (EDGE) and ranks (EDGE_ranks), their HEDGE scores (HEDGE) and ranks (HEDGE_rank) and their LEDGE scores (LEDGE) and ranks (LEDGE_rank).

File Name: Supplementary Data 2
Description: List of the 1369 TOP 25% HEDGE species (species) ranked by their HEDGE rank (HEDGE_rank), their HEDGE score (HEDGE_score) and their conservation measures at the first level of classification (conservation_classification).

File Name: Supplementary Data 3
Description: List of the 1369 TOP 25% LEDGE species (species) ranked by their LEDGE rank (LEDGE_rank), their LEDGE score (LEDGE_score) and their conservation measures at the first level of classification (conservation_classification).
